# The relationship between GSTA1, GSTM1, GSTP1, and GSTT1 genetic polymorphisms and bladder cancer susceptibility: A meta-analysis

**DOI:** 10.1097/MD.0000000000004900

**Published:** 2016-09-16

**Authors:** Yajie Yu, Xiao Li, Chao Liang, Jingyuan Tang, Zhiqiang Qin, Chengming Wang, Weizhang Xu, Yibo Hua, Pengfei Shao, Ting Xu

**Affiliations:** a Department of Urology, The First Affiliated Hospital of Nanjing Medical University; b Department of Thoracic Surgery, Nanjing Medical University Affiliated Cancer Hospital; Jiangsu Key Laboratory of Molecular and Translational Cancer Research, Cancer Institute of Jiangsu Province; c Department of Urologic Surgery, The Affiliated Cancer Hospital of Jiangsu Province of Nanjing Medical University, Nanjing, China.

**Keywords:** bladder cancer, glutathione S-transferases, meta-analysis, single gene polymorphism, susceptibility

## Abstract

**Background:**

Previous studies have investigated the relationship between GSTA1, GSTM1, GSTP1, and GSTT1 polymorphisms and bladder cancer (BCa) susceptibility, respectively, but the results remain inconsistent. So, we conducted this meta-analysis including 79 case–control studies to explore such relationships.

**Methods:**

We searched PubMed, EMBASE, Cochrane library, Web of Science, and CNKI for relevant available studies. The pooled odds ratios (ORs) with 95% confidence intervals (CIs) were implemented to evaluate the intensity of associations. Publication bias was estimated using Begg funnel plots and Egger regression test. To assess the stability of the results, we used sensitivity analysis with the method of calculating the results again by omitting 1 single study each time. Between-study heterogeneity was tested using the *I*^2^ statistic.

**Results:**

No significant association between GSTA1 polymorphism and BCa susceptibility (OR = 1.05, 95% CI 0.83–1.33) was noted. Besides, meaningful association between individuals who carried the GSTM1 null genotype and increased BCa risk was detected (OR = 1.39, 95%CI 1.28–1.51). When stratified by ethnicity, significant difference was found in both Caucasian (OR = 1.39, 95% CI 1.23–1.58) and Asian populations (OR = 1.45, 95% CI 1.31–1.61). Moreover, in the subgroup analysis by source of controls (SOC), the results were significant in both hospital-based control groups (OR = 1.49, 95% CI 1.35–1.64) and population-based control groups (OR = 1.21, 95% CI = 1.07–1.37). Additionally, the analysis revealed no significant association between GSTP1 polymorphism and BCa risk (OR = 1.07, 95% CI 0.96–1.20). What is more, significant associations between GSTT1 polymorphism and BCa susceptibility were discovered (OR = 1.11, 95% CI 1.00–1.22). In the subgroup analysis by ethnicity, significant associations between GSTT1 null genotype and BCa risk were observed only in Caucasians (OR = 1.25, 95% CI 1.09–1.44). Furthermore, when stratified by SOC, no obvious relationship was found between the GSTT1 null genotype polymorphism with hospital-based population (OR = 1.11, 95% CI 0.97–1.28) or population-based population (OR = 1.10, 95% CI 0.96–1.27).

**Conclusion:**

This study suggested that GSTM1 null genotype and GSTT1 null genotype might be related to higher BCa risk, respectively. However, no associations were observed between GSTA1 or GSTP1 polymorphisms and BCa susceptibility.

## 1 Introduction

Bladder cancer (BCa), with an increasing incidence and mortality nowadays, has become the 9th most common cancer and the 14th leading cause of death due to cancer worldwide.^[1]^ An estimated 429,800 new cases of BCa and 165,100 deaths took place in 2012 worldwide.^[2]^ As a complicated and multifactorial procedure, the initiation and development of BCa are still not completely understood.^[3]^ However, the risk factors could be mainly classified into 3 subgroups: long-term inflammation stimulation, specific chemical exposure, and genetic factors.^[4]^ Interestingly, some people never get BCa even though exposed to specific chemicals. In contrast, many BCa patients do not have those known risk factors, suggesting that genetic factors might play a significant role in bladder carcinogenesis.^[5,6]^

Glutathione S-transferases (GSTs), existing in almost all living organisms, are members of a polygene family of isoenzymes.^[7]^ GSTs are a family of multifunctional phase II enzymes that catalyze the combination of many exogenous and endogenous electrophilic compounds with glutathione, which are characterized with assisting the detoxification of various therapeutic drugs, carcinogens, products of oxidative stress, toxins, and chemical mutants.^[8,9]^ In humans, GSTs were encoded by 8 different gene families. Among them, 4 are mainly expressed in tissues: GSTA, GSTM, GSTP, and GSTT. Accordingly, the GSTA1, GSTM1, GSTP1, and GSTT1 genes are located at chromosome 6p12.1, 1p13.3, 11q13, and 22q11.23, respectively.

Over the last 2 decades, plentiful studies have been carried out to investigate the association between GSTs and the risk of BCa, but these studies have reported conflicting results. A single study might fail to demonstrate the complicated genetic relationship due to small sample size, but meta-analysis could increase the statistical power through detecting overall effects. Previously, meta-analysis has been performed to find out the relationship between GSTM1, GSTP1, GSTT1, and BCa, respectively.^[10–14]^ Although the results remain inconclusive or even contradictory. In addition, the relationship between GSTA1 and BCa susceptibility has not been qualitatively studied before. Some related case–control studies have been released after the previous meta-analyses, which may generate influence on the conclusions. Therefore, we conducted such meta-analysis to assess these relationships by including all eligible articles.

## 2 Materials and methods

### 2.1 Search strategy

We did a systematic search of PubMed, EMBASE, Cochrane library, Web of Science, and CNKI up to December 2015 by using the combination of the following key words: “glutathione S-transferase A1” or “GSTA1,” “glutathione Stransferase M1” or “GSTM1,” “glutathione S-transferase P1” or “GSTP1,” “glutathione S-transferase T1” or “GSTT1,” “bladder” or “urothelial,” “cancer” or “carcinoma” or “neoplasm,” and “polymorphism” or “polymorphisms” without any restriction on language. The reference lists of the selected papers were searched by hand for potentially eligible articles. We only included the study with the most recent and/or the largest sample size when several studies had partially overlapped or similar data.

### 2.2 Selection criteria

For this meta-analysis, the inclusion criteria were as follows: case–control studies with the original date for the evaluated associations between GSTA1, GSTM1, GSTP1 and/or GSTT1 polymorphisms, and BCa risk; the diagnosis of the patients with BCa was confirmed pathologically, and the controls were confirmed free of any cancer; and sufficient published data about the size of the sample, odds ratio (OR), and their 95% confidence interval (CI). The exclusion criteria were duplicates of previous publication; no control subjects; and patients without confirmation of BCa or mixed with other diseases.

If study populations were the same or duplicate data were published, only the study with the largest number of sample size was included. We did not need to obtain ethical approval or informed consent because our data were extracted from previous studies. Nevertheless, the included studies in our review did get patient consent, and each study was approved by an ethics committee.

### 2.3 Data extraction

Data were independently extracted from all eligible publications by 5 investigators (YJY, XL, CL, JYT, and ZQQ), and quality assessment was conducted by 3 authors (YJY, XL, and CL). When meeting conflicting opinions about inclusion, disagreements were resolved by discussion among team members. Relevant data were extracted from each eligible study and carefully recorded, including involved genes, 1st author name, year of publication, the ethnicity of the study population, subject source, total number of cases and controls, and different number of genotypes in cases and controls. If important unpublished information were needed, we also e-mailed the original authors. According to source of controls (SOC), studies were classified into hospital-based (HB) and population-based (PB) groups. Ethnic groups were principally defined as Caucasian, Asian, African, or Mixed.

### 2.4 Statistical analysis

ORs with 95% CIs were implemented. The heterogeneity was estimated using the χ^2^-based Q statistic, and heterogeneity was considered statistically significant when *P* heterogeneity ≤0.1 or *I*^2^ > 50%.^[15]^ If the presence of heterogeneity was found, the random-effects model would be utilized. Otherwise, fixed-effects model would be performed. Then, subgroup analysis was further carried out by ethnicity and SOC properly.

To assess the stability of the results, we used sensitivity analysis with the method of calculating the results again by omitting 1 single study each time.^[16]^ To check the publication bias between the studies, Egger linear regression test and Begg funnel plots were executed.^[17]^ Hardy–Weinberg equilibrium was assessed by the goodness-of-fit Chi-square test, and *P* < 0.05 was considered as an obviously selective bias.^[18]^ All statistical analyses tests were performed with Stata software (version 12.0; Stata Corp LP, College Station, TX). All *P* values below 0.05 were considered statistically significant.

## 3 Results

### 3.1 Literature search and studies characteristics

**Figure 1:**
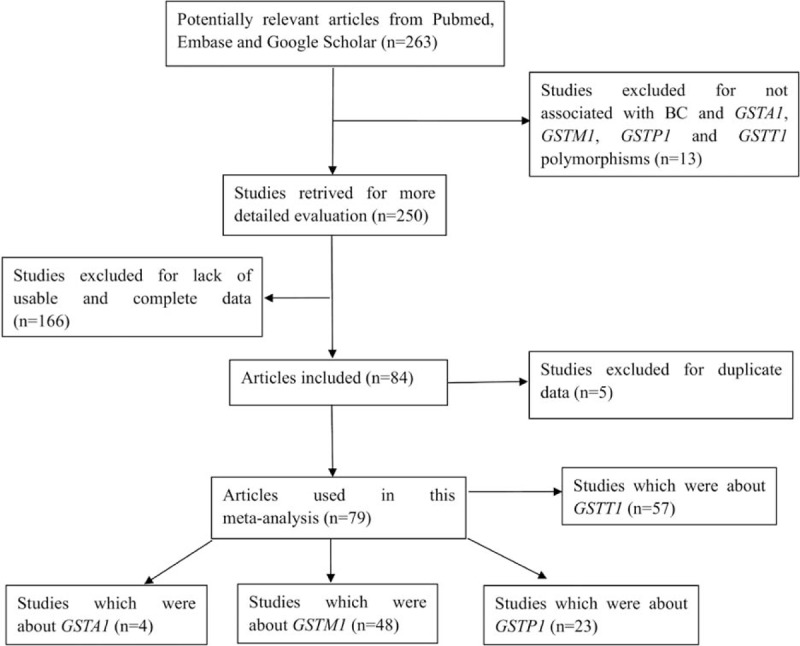
Flowchart of literature search and selection process.

Figure 1 shows the flowchart of literature search and selection process. Finally, a total of 79 case–control studies were included according to the inclusion criteria.^[19–97]^ Characteristics of individual study qualified for the current meta-analysis (GSTA1, GSTM1, GSTP1, and GSTT1, respectively) are presented in Tables 1–4 individually. This meta-analysis results of association between GSTs polymorphism and BCa risk are shown in Table 5.

**Table 1:**
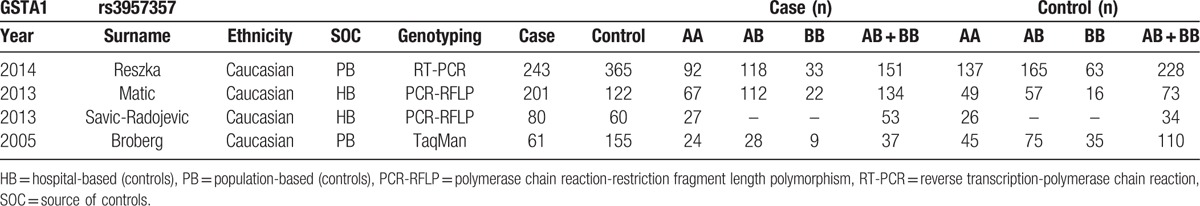
Characteristics of individual studies included in the meta-analysis.

**Table 2:**
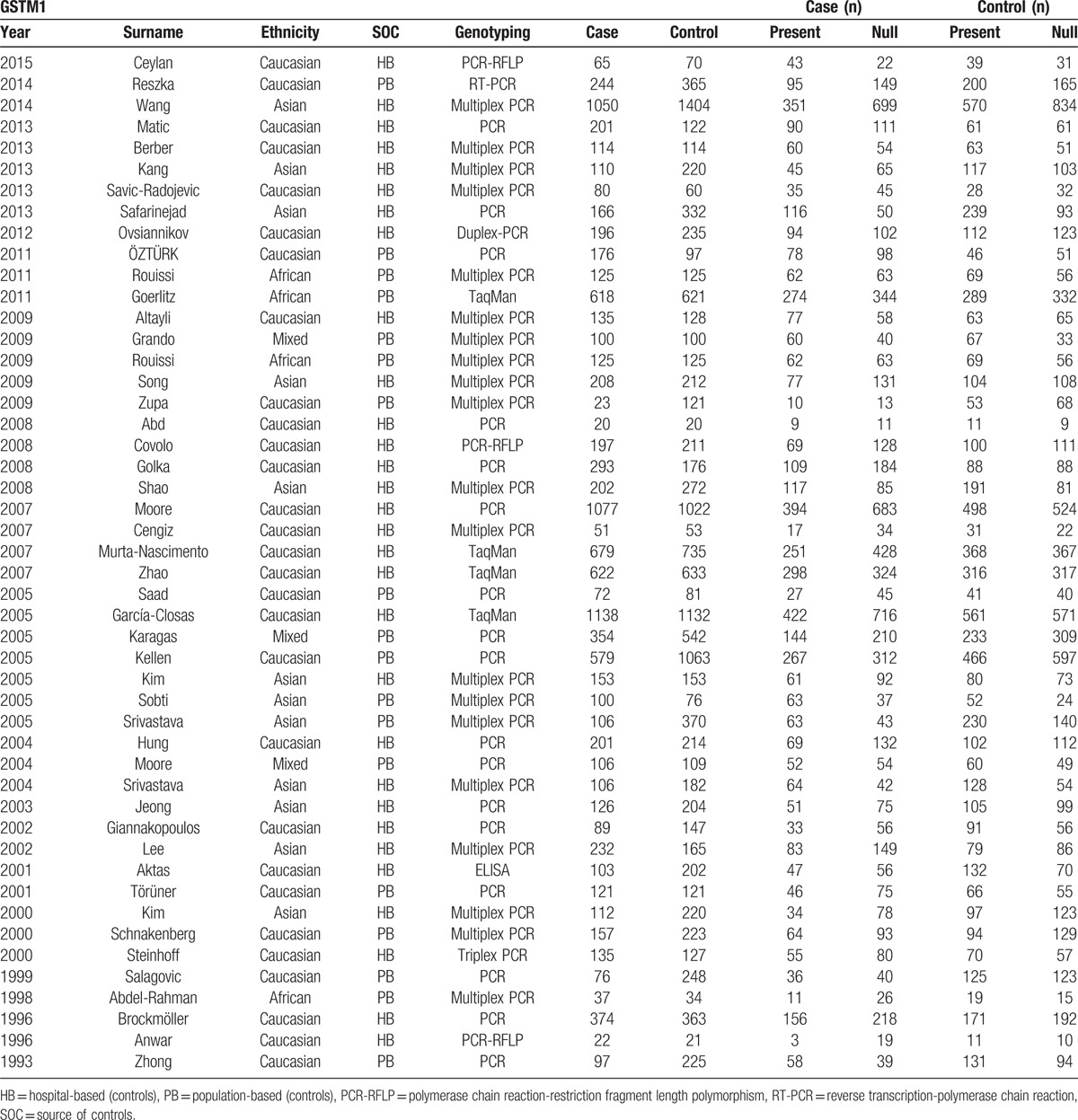
Characteristics of individual studies included in the meta-analysis.

**Table 3:**
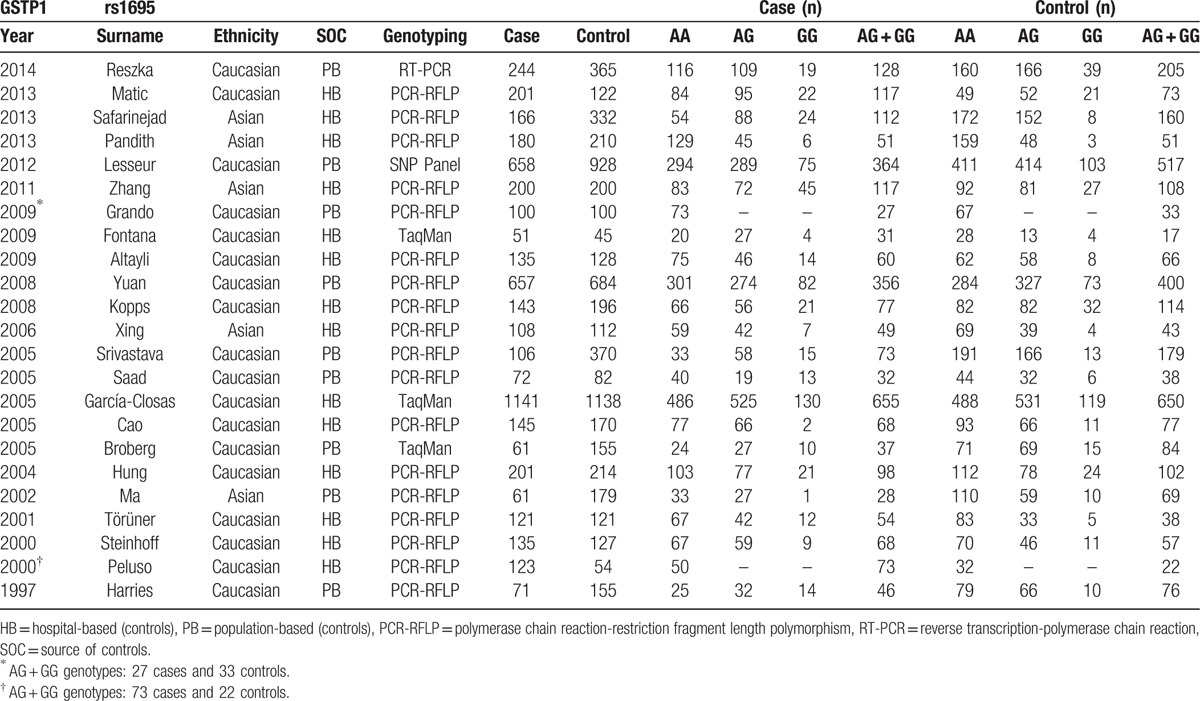
Characteristics of individual studies included in the meta-analysis.

**Table 4:**
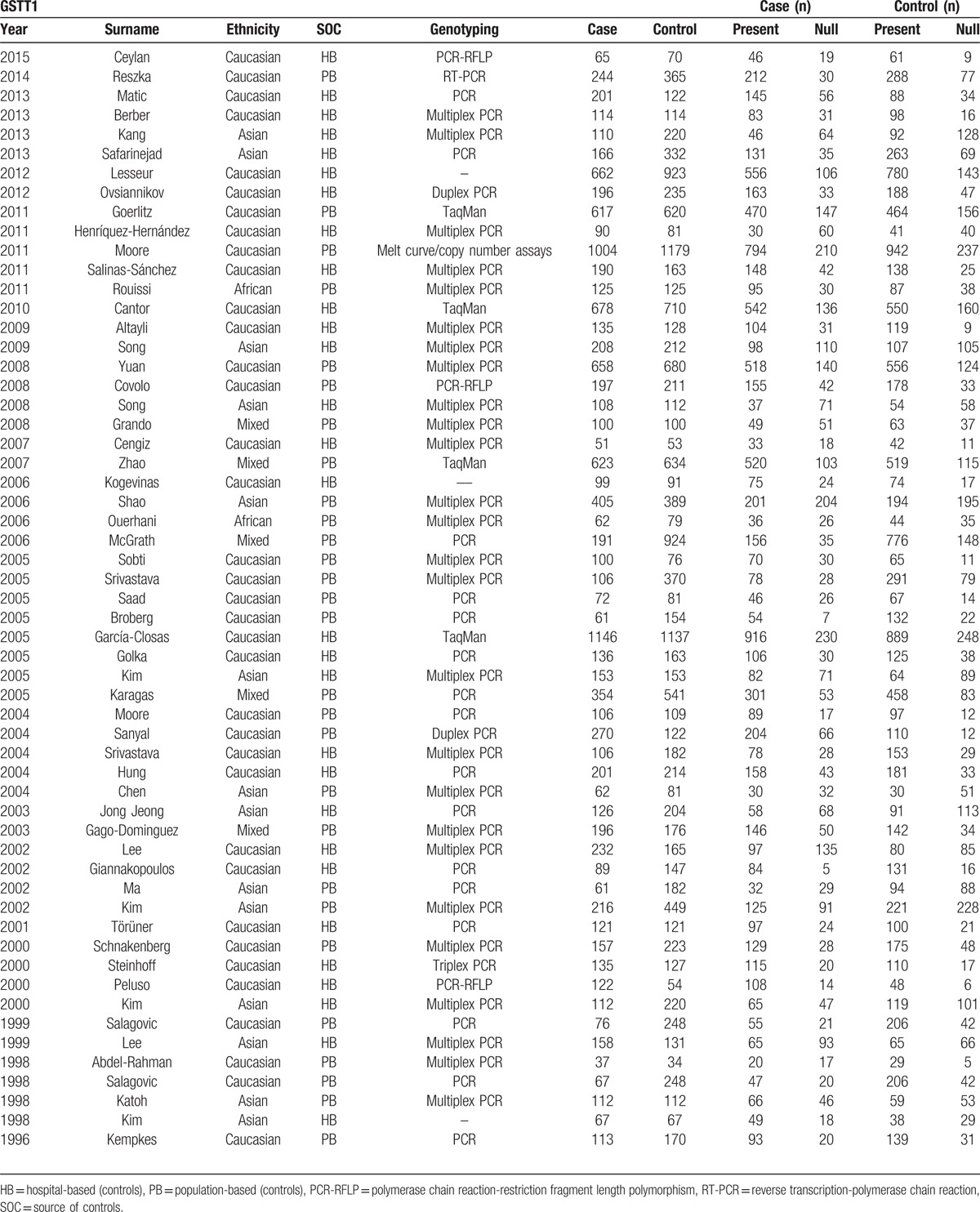
Characteristics of individual studies included in the meta-analysis.

**Table 5:**
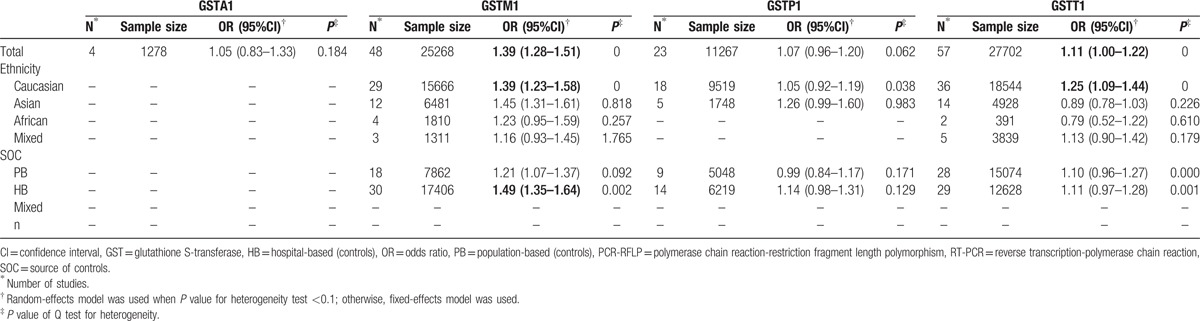
Meta-analysis results of association between GSTs polymorphism and bladder cancer risk.

### 3.2 GSTA1

**Figure 2:**
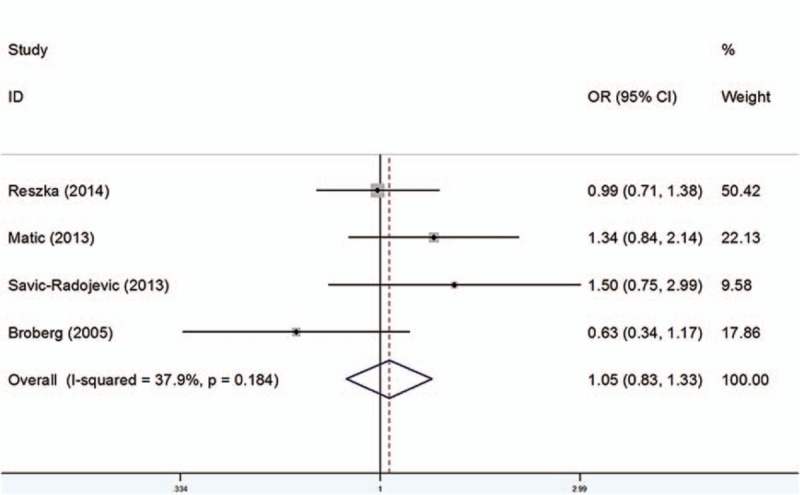
Forest plots of the association between GSTA1 polymorphism and bladder cancer susceptibility. CI = confidence interval, OR = odds ratio.

Four studies consisting of 585 cases and 702 controls were adopted in order to evaluate the relationship between GSTA1 polymorphism and BCa risk. As shown in Fig. 2, the results indicated no significant association between GSTA1 polymorphism and BCa susceptibility (OR = 1.05, 95% CI 0.83–1.33). Subgroup analysis was not performed owing to the limited studies.

### 3.3 GSTM1

**Figure 3:**
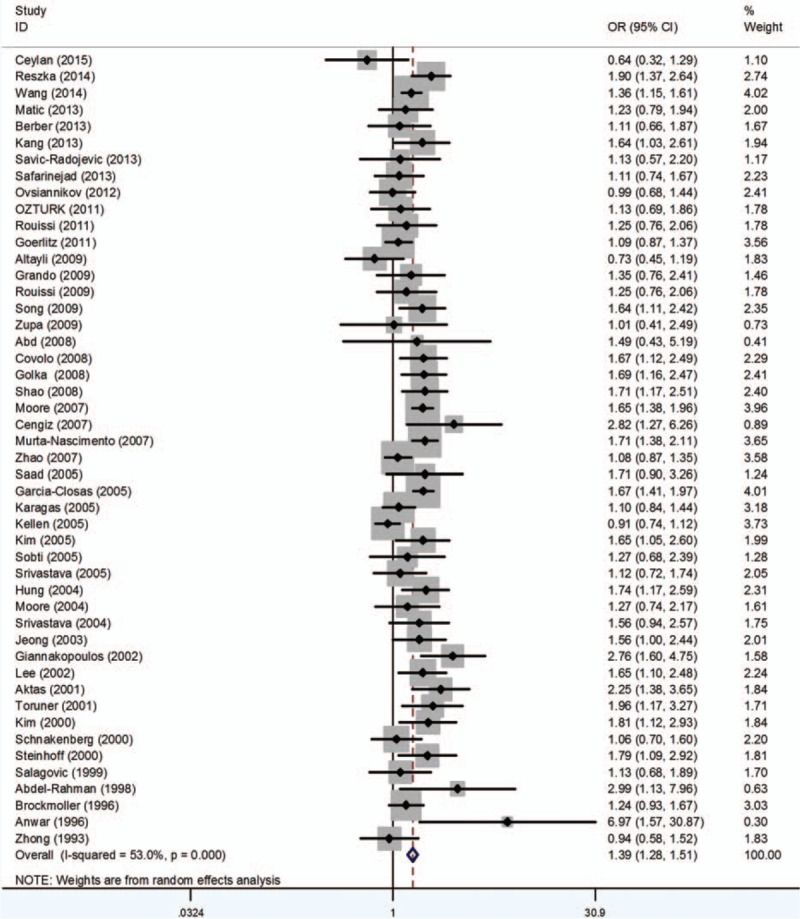
Forest plots of the association between GSTM1 polymorphism and bladder cancer susceptibility. CI = confidence interval, OR = odds ratio.

As shown in Table 5, 48 studies including 11,473 cases and 13,795 controls were analyzed. Overall, significant associations between individuals who carried GSTM1 null genotype and increased BCa risk were observed (OR = 1.39, 95%CI 1.28–1.51) (Fig. 3). When stratified by ethnicity, significant difference was detected in Caucasian (OR = 1.39, 95% CI 1.23–1.58) and Asian populations (OR = 1.45, 95% CI 1.31–1.61) instead of African (OR = 1.23, 95% CI 0.95–1.59) or Mixed populations (OR = 1.16, 95% CI 0.93–1.45). In addition, in the subgroup analysis by SOC, the results were significant both in HB populations (OR = 1.49, 95% CI 1.35–1.64) and PB populations (OR = 1.21, 95% CI 1.07–1.37).

### 3.4 GSTP1

**Figure 4:**
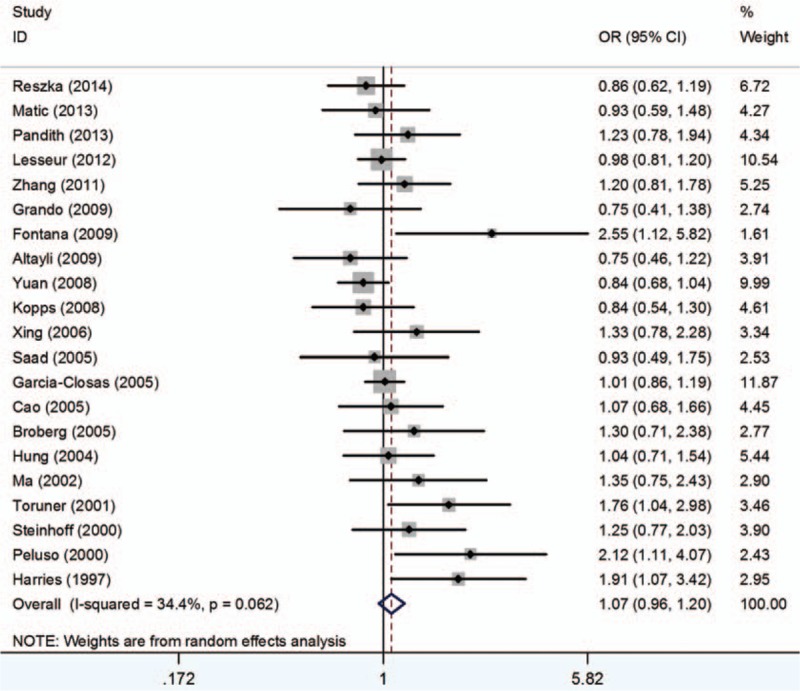
Forest plots of the association between GSTP1 polymorphism and bladder cancer susceptibility. CI = confidence interval, OR = odds ratio.

Twenty-three studies involving 5080 cases and 6187 controls were included in this study. Because a few studies provided precise data of genotypes, only dominant model could be carried out with all studies. Generally, the analysis revealed no significant association between GSTP1 Ile105Val polymorphism and BCa risk (OR = 1.07, 95% CI 0.96–1.20) (Fig. 4). No significant relationship was observed between GSTP1 polymorphism and BCa risk in patients when stratified by ethnicity. Meanwhile, there seems no relationship between GSTP1 polymorphism and the susceptibility of BCa when stratified by SOC (Table 5).

### 3.5 GSTT1

**Figure 5:**
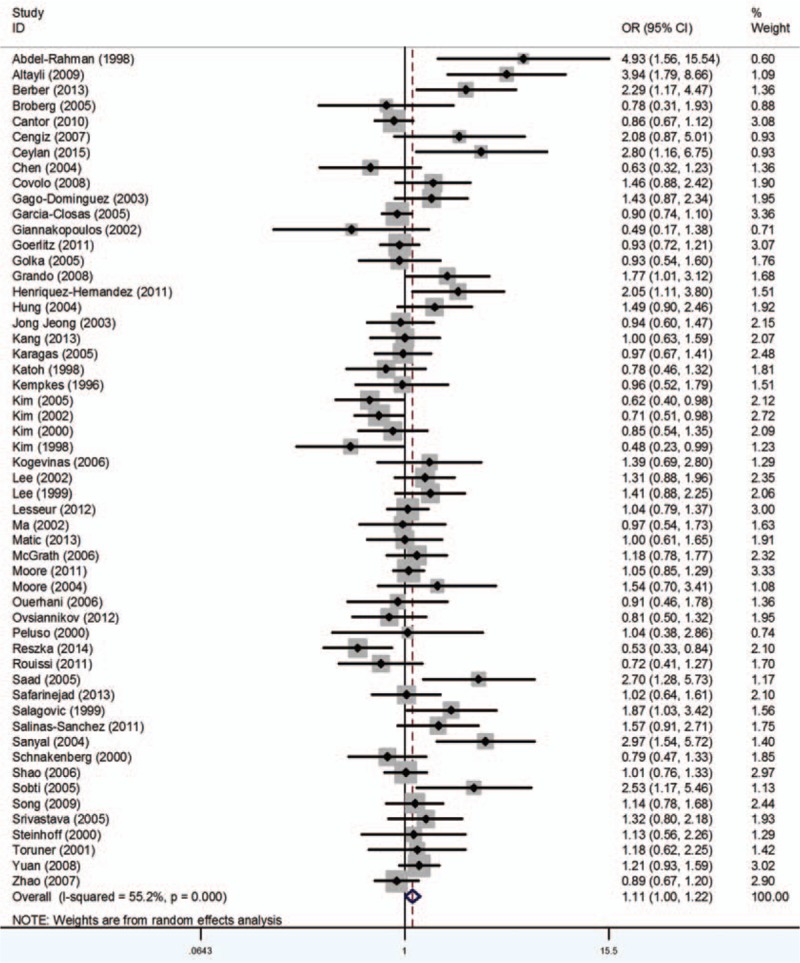
Forest plots of the association between GSTT1 polymorphism and bladder cancer susceptibility. CI = confidence interval, OR = odds ratio.

Fifty seven studies including 12,369 cases and 15,333 controls were analyzed. The results indicated significant association between GSTT1 polymorphism and BCa susceptibility (OR = 1.11, 95% CI 1.00–1.22) (Fig. 5). In the subgroup analysis by ethnicity, significant associations between GSTT1 null genotype and BCa risk were noted only in Caucasians (OR = 1.25, 95% CI 1.09–1.44). Additionally, when stratified by SOC, no obvious relationship was detected between the GSTT1 null genotype polymorphism with HB (OR = 1.11, 95% CI 0.97–1.28) or PB (OR = 1.10, 95% CI 0.96–1.27), respectively (Table 5).

### 3.6 Sensitivity analysis

Sensitivity analysis was utilized to identify the influence of each study on the pooled OR by consecutively omitting 1 study each time for all subjects and subgroups. The sensitivity analysis for GSTA1, GSTM1, GSTP1, and GSTT1 polymorphism showed that no individual study affected the pooled OR significantly, which indicated that our results were reliable.

### 3.7 Publication bias

The publication bias of studies GSTA1, GSTM1, GSTP1, and GSTT1 were assessed, respectively, using Begg and Egger funnel plot. The overall outcomes revealed that our results were statistically dependable.

## 4 Discussion

BCa is one of the most common cancers of the urinary tract. However, the exact mechanisms of bladder carcinogenesis remain unclear. There is a growing realization that the development of BCa is caused by a complex interaction of both genetic and environmental factors.^[98]^ Although genetic factors are considered to be a crucial part of the pathogenic process of BCa, especially the polymorphisms in metabolic pathways.^[99]^ As one of the most important parts of phase II super family of metabolism enzymes, GSTs are composed of 7 classes (α, μ, ω, π, σ, θ, ξ).^[100]^ Among them, GSTA1, GSTM1, GSTP1, and GSTT1 are considered to be the most important. Almost all members of the GST family show genetic polymorphism, which leads to a complete absence or lowering of enzyme activity.

GSTA1 has 3 single nucleotide polymorphisms (SNPs): −567TOG, −69COT, and −52GOA.^[101]^ Differential expression with lower transcriptional activation of variant GSTA1∗B (−567G, −69T, and −52A) than common GSTA1∗A allele (−567T, −69C, and −52G) are resulted from these replacements.^[102]^ GSTM1 plays an important role in preventing the development of cancers. The inherited homozygous absence of the GSTM1 gene results in the deficiency of the enzyme activity.^[103]^ GSTP1 is an important part of GST families, and the most commonly studied GSTP1 variant is exon 5 Ile105Val, encoding an Ile/Val exchange at codon 105 (Ile105Val; A105G) (rs947894), which has been shown to be linked to lower expression of metabolic activity.^[104]^ People with the GSTT1 null genotype was reported to have decreased enzyme activity and decreased ability to detoxify the environmental and dietary agents, especially 1,3-butadiene and ethylene oxide, which could induce chromosomal damage and make people more susceptible to cancer.^[105]^ By catalyzing the detoxification of electrophilic compounds through conjugation with glutathione, these enzymes can prevent cells from damage.^[106]^ Besides, GSTs are able to regulate the induction of other proteins and enzymes which is important for cellular functions. The polymorphisms affect the enzyme activity, leading to increased genotoxic damage and affect the transportation of steroid hormones, causing the development of cancer eventually.^[107,108]^ GSTs are essential for maintaining genomic integrity because electrophilic compounds could damage the DNA.^[109]^ Therefore, GSTA1, GSTM1, GSTP1, and GSTT1 may play an important role in the development of BCa.

Recently, there were increasing case–control studies concerned with the associations between GSTs polymorphisms and BCa susceptibility.^[19–97]^ Nevertheless, the inconsistent results of them might owe to limited sample size, various methodologies, and race and dissimilar source of controls. Although several meta-analyses have explored the relationship between GSTM1, GSTP1, and GSTT1 polymorphisms and BCa susceptibility, respectively,^[10–14]^ the results remain unclear. Besides, because of relatively small number of studies, no meta-analysis on GSTA1 has been performed before. What is more, additional studies have been published since the last meta-analysis. So, all these might have generated great influence to the previous conclusions. Thus, we did this meta-analysis.

For the first time, we performed meta-analysis on the relationship between GSTA1 polymorphism and BCa susceptibility. We included 4 case–control studies in this meta-analysis, and the results suggested that there was no association. According to the published papers, the conclusion on the relationship between GSTA1 polymorphism and BCa susceptibility is inconsistent. The exact mechanism of the influence of GSTA1 polymorphism on BCa is still unclear. However, in association with smoking, low activity GSTA1 seems to increase individual susceptibility to BCa.^[20]^ The limited amount of involved studies may become a major factor which could influence the evaluation of the real association between GSTA1 polymorphism and BCa risk.

The analysis of the present studies indicated that the null genotype of GSTM1 polymorphism significantly increases BCa susceptibility. Jiang et al^[10]^ performed a meta-analysis indicating the similar results with ours in 2011, which included 33 studies. Nevertheless, 48 studies were involved in our meta-analysis, which could provide more comprehensive and reliable results.

Meanwhile, similar to the outcome of the meta-analysis conducted by Gong et al in 2012,^[14]^ significant associations between GSTT1 polymorphism and BCa susceptibility were discovered. However, we included 7 more studies, which could be more credible.

In the aspect of GSTP1, contrary to the previous meta-analysis (Wang et al^[13]^ and Kellen et al^[111]^), this analysis revealed no significant relationship between GSTP1 polymorphism and BCa risk. Wang et al internalized 25 studies, but there were 2 duplicates of previous publication, so we excluded them. Although the result changes, we think it is more believable. And Kellen et al included 16 studies (4273 cases and 5081 controls), whereas we selected 23 studies involving 5080 cases and 6187 controls. Additional studies have been published since the last meta-analysis, which would might change the previous results. So, we think the result of our study is more reliable. More studies are required to validate our results.

Alternatively, subgroup analysis was performed according to ethnicity in GSTM1, GSTP1, or GSTT1 genetic variants. For GSTM1, when stratified by ethnicity, significant difference was detected in both Caucasian and Asian populations instead of African populations and Mixed populations. No significant relationship was observed between GSTP1 polymorphism and BCa risk in patients when stratified by ethnicity. For GSTT1, significant associations were observed only in Caucasians. As a complicated multigenetic disease, cancer has diversity among different ethnic populations, and the existence of the discrepancy might owing to different genetic background.^[110]^ As a result of ethnic differences, the incidence of gene polymorphisms may vary notably among different phyletic populations. Although the possible reasons of the conflicting results were unknown, there might be several explanations for it. First, among different ethnic groups, various environmental factors and genetic backgrounds might not be exposed sheerly, which might also be affected by unidentified genes. Second, the selection bias and limitation of sample size should also be taken into consideration.

In the present meta-analysis, the cases and controls were from dissimilar sources. The results suggest that there is association between GSTM1 null genotype and BCa susceptibility both in the subgroup analysis of studies with HB and PB controls. Our meta-analysis also revealed there is no relationship between other GSTs polymorphisms and BCa risk in their respective SOC groups. Subgroup analysis was not performed owing to the limited studies for GSTA1. However, more prospective studies should be performed to evaluate if there has indeed an association between the other GSTs polymorphisms and BCa risk exists in the subgroup analysis of SOC.

Despite the certain conclusions generated in this study, there still exist several limitations. First, the sample sizes of GSTM1, GSTP1, and GSTT1 were large enough, nevertheless which caused possible false positive conclusions. Second, the number of some subgroups was relatively small, and it is hard to search for the reliable association with limited statistical power. Third, when it comes to GSTA1 polymorphism, the sample size was too small. Additional studies with higher quality and larger sample size should be included in the future to verify our result. Fourth, BCa results from complex interactions between a variety of genetic and environmental factors, thereby suggesting that BCa susceptibility would not be influenced by any single gene. BCa is a multifactorial disease, so the complex interactions like gene-environment factors could not be ignored. Last, the total outcomes were based on unadjusted effect estimates without enough data for the adjustment by other covariates, such as smoking status, age, gender, and so on. The influence of confounding factors should be payed more attention. Hence, a more precise meta-analysis could be conducted if detailed data of some individual studies can be accessed.

The results indicated that the GSTM1 null genotype might elevate BCa susceptibility, and the GSTT1 polymorphism might enhance BCa risk. No significant associations were observed between GSTA1 or GSTP1 polymorphism and BCa risk. For the 1st time, we performed this meta-analysis to evaluate the association between GSTA1 polymorphism and BCa risk. However, taking the restriction of sample size into consideration, analysis with larger and more well-designed studies is required to validate our results. In the future, the analysis of different combinations of polymorphisms of the 4 isoforms could be performed if the data is available.
